# Targeting Neural Oscillations for Cognitive Enhancement in Alzheimer’s Disease

**DOI:** 10.3390/medicina61030547

**Published:** 2025-03-20

**Authors:** Federica Palacino, Paolo Manganotti, Alberto Benussi

**Affiliations:** Neurology Unit, Department of Medical, Surgical and Health Sciences, University of Trieste, 34149 Trieste, Italy; federicapalacino@gmail.com (F.P.); pmanganotti@units.it (P.M.)

**Keywords:** neural oscillations, cognitive enhancement, brain stimulation, rTMS, tDCS, tACS, tFUS

## Abstract

Alzheimer’s disease (AD), the most prevalent form of dementia, is marked by progressive cognitive decline, affecting memory, language, orientation, and behavior. Pathological hallmarks include extracellular amyloid plaques and intracellular tau tangles, which disrupt synaptic function and connectivity. Neural oscillations, the rhythmic synchronization of neuronal activity across frequency bands, are integral to cognitive processes but become dysregulated in AD, contributing to network dysfunction and memory impairments. Targeting these oscillations has emerged as a promising therapeutic strategy. Preclinical studies have demonstrated that specific frequency modulations can restore oscillatory balance, improve synaptic plasticity, and reduce amyloid and tau pathology. In animal models, interventions, such as gamma entrainment using sensory stimulation and transcranial alternating current stimulation (tACS), have shown efficacy in enhancing memory function and modulating neuroinflammatory responses. Clinical trials have reported promising cognitive improvements with repetitive transcranial magnetic stimulation (rTMS) and deep brain stimulation (DBS), particularly when targeting key hubs in memory-related networks, such as the default mode network (DMN) and frontal–parietal network. Moreover, gamma-tACS has been linked to increased cholinergic activity and enhanced network connectivity, which are correlated with improved cognitive outcomes in AD patients. Despite these advancements, challenges remain in optimizing stimulation parameters, individualizing treatment protocols, and understanding long-term effects. Emerging approaches, including transcranial pulse stimulation (TPS) and closed-loop adaptive neuromodulation, hold promise for refining therapeutic strategies. Integrating neuromodulation with pharmacological and lifestyle interventions may maximize cognitive benefits. Continued interdisciplinary efforts are essential to refine these approaches and translate them into clinical practice, advancing the potential for neural oscillation-based therapies in AD.

## 1. Introduction

Alzheimer’s disease (AD) is the most common form of dementia worldwide [1]. It is clinically characterized by a gradual cognitive decline, beginning with memory loss and progressively affecting various cognitive domains. These include difficulties in communication and language, disorientation to time and place, becoming lost even in familiar settings, and changes in mood, behavior, vigilance, or levels of consciousness [2]. The pathological hallmarks of AD consist of extracellular amyloid plaques (formed from the abnormal cleavage of amyloid precursor protein—APP) and intracellular neurofibrillary tangles composed of hyperphosphorylated tau (p-tau). These pathological changes arise from a multifactorial etiology driven by the interplay of genetic predispositions and environmental influences [3].

This pathological substrate triggers a cascade of oxidative and neuroinflammatory damage, including microglial activation, astrogliosis, neurotrophic factor deficits, mitochondrial dysfunction, impaired amyloid clearance, and brain atrophy [4]. These processes ultimately disrupt synaptic activity, which not only contributes to but is also influenced by the pathological underlying pathology, leading to neuronal dysfunction and connectivity loss [5,6]. Synaptic loss is considered an early event in AD pathogenesis, occurring even before significant neuronal degeneration and playing a critical role in disrupting neuronal function.

AD neuropathogenesis can be conceptualized at three levels: (1) the microscale, involving genetic mutations and molecular aberrations; (2) the mesoscale, characterized by alterations in homeostasis, clearance mechanisms, and neuroimmunity; and (3) the macroscale, reflected in circuit dysfunction. Brain oscillations, defined as rhythmic patterns arising from synchronized neural activity, are crucial for cognition and neural computations. These oscillations, categorized by frequency bands, become disrupted in AD, often years before the onset of clinical symptoms [7].

Brain oscillations are intimately linked to brain states, such as levels of consciousness, vigilance, wakefulness, and arousal. These states range from full concentration and conscious awareness to inattentiveness, drowsiness, and deeper stages of sleep [8]. Importantly, brain states do not represent a continuous functional gradient but instead reflect distinct computational modes of brain activity, determined by changes in neural synchrony. Neural synchrony refers to the correlation of neural activity within or across neuronal populations. During resting or non-active states, such as slow-wave sleep or quiet wakefulness, neuronal activity is predominantly characterized by synchronized, slow, high-amplitude oscillations [9,10]. Increased synchrony among neurons, manifesting as slow-frequency and high-amplitude fluctuations, serves to deactivate specific brain regions. Conversely, the suppression of high-amplitude fluctuations and the desynchronization of neuronal activity prepare neurons to respond to inputs.

The relationship between the degree of correlated neuronal activity and brain functional states is most evident at the level of networks and circuits. In AD, the normal rhythmic oscillatory activity, and specifically the functional dynamics at the network level, which are crucial for memory encoding and retention, are impaired. These impairments include deficits in the activation and deactivation of specific networks, as well as hypersynchrony within these networks.

In recent years, significant efforts have been devoted to targeting and modulating these mechanisms to improve brain function in AD. Both invasive and non-invasive brain stimulation techniques have shown therapeutic potential, demonstrating efficacy in enhancing cognitive functions or slowing cognitive decline in AD patients. These interventions primarily target key hubs within the frontoparietal network and the default mode network, aiming to restore their functional integrity.

This work presents a comprehensive narrative review summarizing key findings from preclinical and clinical studies on neural oscillations as therapeutic targets in AD. The aim of this review is to synthesize current evidence on the physiological roles of neural oscillations, their pathological alterations in AD, and their potential modulation through neuromodulatory interventions. We examine preclinical and clinical findings on brain stimulation techniques, including deep brain stimulation, repetitive transcranial magnetic stimulation, transcranial direct current stimulation, transcranial alternating current stimulation, transcranial pulse stimulation, ultrasound stimulation, sensory stimulation, and photobiomodulation. By summarizing the strengths, limitations, and future directions of neuromodulation in AD, this review aims to provide a comprehensive resource for researchers and clinicians in the field. The literature search was conducted across the PubMed, Scopus, and Web of Science databases, focusing on publications between 2000 and 2024, with particular attention paid to recent advancements in the field of neural oscillations, brain stimulation techniques, and Alzheimer’s disease. Keywords included “Alzheimer’s disease”, “neural oscillations”, “brain stimulation”, “repetitive transcranial magnetic stimulatio”, “transcranial alternating current stimulation”, “transcranial pulse n”, “transcranial direct current stimulationstimulation”, “ultrasound stimulation”, “sensory stimulation”, “photobiomodulation”, “neuromodulation”, and “EEG”. Additional references were identified through manual searches of relevant review articles and meta-analyses. Only peer-reviewed studies written in English were considered for inclusion.

## 2. Neural Oscillations: Mechanisms and Relevance to Cognition

Oscillatory electrical activity is a defining feature of most neuronal networks. To understand how oscillations contribute to higher brain functions, it is essential to first consider their underlying mechanisms. A key requirement for the generation of network oscillations is regular and synchronized neuronal activity. Single neurons possess complex dynamics that allow them to resonate at multiple frequencies, ranging from 0.05 Hz to 500 Hz. These frequencies are categorized into distinct bands, calculated using a constant ratio between neighboring frequencies on a natural logarithmic scale of their mean frequencies [11].

When a neuron fires action potentials in a rhythmic manner, it induces a periodic fluctuation in the intracellular membrane potential of its postsynaptic target cells. If multiple neurons fire action potentials both rhythmically and synchronously, the resulting output signal is amplified, creating temporal windows of increased and reduced excitability in a larger population of target cells. This rhythmic synaptic activation pattern produces a fluctuating field potential signal, which represents the sum of the synchronous oscillatory activity from a large number of favorably oriented pyramidal cortical neurons. These neurons predominantly receive dendritic postsynaptic signals from the thalamus, other cortical modules, or the cholinergic systems of the midbrain [12,13].

The thalamus serves as the primary pacemaker of global oscillatory brain activity, modulating cortical neurons via positive or negative feedback loops. This modulation occurs through two distinct firing patterns: tonic firing, which is characteristic of wakefulness and dreaming mental activity, and rhythmic discharge, typical of deep sleep. The thalamic firing pattern is determined by resting membrane potentials, the activation of calcium channels, and inputs from the cortex or cholinergic nuclei of the brainstem.

The divergence of synaptic connections promotes a high level of spatial coherence between different brain regions, enabling them to resonate at similar frequencies, an essential feature of network oscillations. By analyzing the power density (i.e., the square of the wave amplitude over time) of local field potentials, it is possible to infer the coherence spectrum and the phase signal interdependencies between distant brain regions. High interdependence indicates strong functional connectivity among regions within the same cortical network.

In summary, oscillations emerge from the dynamic interplay of intrinsic cellular properties and circuit interactions. Each frequency band is associated with specific brain states within neuronal networks. Neighboring frequency bands within the same network often reflect different brain states and may compete with each other. However, multiple rhythms can coexist within the same or different structures, interacting dynamically to influence brain function [14,15].

### 2.1. Alpha Rhythm

The alpha rhythm, historically referred to as “Berger’s waves”, represents oscillations in the frequency range of 8–13 Hz. It is predominantly recorded from the occipital cortex during relaxed wakefulness with closed eyes [16]. The amplitude of the alpha waves increases during drowsiness and decreases with eye opening or the onset of sleep. The amplitude of alpha waves in a state of quiet wakefulness and their reduction during phase transitions serves as indicators of two key brain functions, namely (1) the ability to maintain cortical inhibition and (2) the brain’s readiness to enter an information-processing state [17].

Alpha oscillations have been identified as the dominant rhythm in the coherence spectrum between the visual cortex and the thalamus, specifically the pulvinar and lateral geniculate nucleus. These findings indicate that the thalamocortical networks play a critical role in generating alpha rhythms and mediating cerebral state transitions [18]. Furthermore, alpha activity has a significant functional role in task performance. Acting as a marker of cortical inhibition, alpha power decreases in areas engaged in task-related processing and increases in regions irrelevant to the task at hand. This modulation of alpha activity has been observed during tasks involving memory load in retention intervals [19,20] where alpha oscillations support the maintenance of information [21,22], as well as during memory-related processes and working memory tasks, reflecting the adaptive role of alpha activity in these cognitive functions [23,24].

### 2.2. Beta Rhythm

The beta rhythm, discovered and named by Hans Berger, encompasses oscillations in the frequency range of 13–30 Hz. It replaces alpha waves with smaller amplitude and faster frequency upon eye opening and is typically associated with normal waking consciousness and task engagement. Beta waves can be further classified into three sub-bands: low-beta waves (Beta1, 13–16 Hz), mid-range beta waves (Beta2, 16.5–20 Hz), and high-beta waves (Beta3, 20.5–30 Hz). Each sub-band is linked to distinct cognitive and behavioral functions.

Low-beta waves (Beta1) are associated with active or busy thinking and heightened states of anxious concentration. Mid-range beta waves (Beta2) are involved in the strengthening of sensory feedback during static motor tasks, the voluntary suppression of movement, and the processing of reward feedback [25,26,27]. High-beta waves (Beta3) are typically observed in the resting state over the lateral prefrontal cortex and demonstrate a postero–anterior increase in frequency, reflecting their role in integrating and modulating cognitive processes [28].

### 2.3. Gamma Rhythm

Gamma rhythm refers to oscillations in the frequency range of 30–100 Hz. Initially detected in the visual cortex of awake monkeys, gamma oscillations have since been observed in the human frontal, parietal, and temporal cortical lobes [29,30,31].

Fast-spiking, parvalbumin-expressing γ-aminobutyric acid (GABA) interneurons, which inhibit neuronal soma, play a central role in generating these oscillations. Gamma rhythms typically arise from the coordinated interaction of excitatory and inhibitory processes across multiple brain regions, including the hippocampus [32].

Neuronal synchronization in the gamma band is most prominent during states of alertness and attentiveness. Often accompanied by a decrease in alpha band activity, gamma synchronization is associated with the initiation of active engagement and processing, involving neurons within the cortico–basal ganglia–thalamo–cortical loop [33]. This circuitry facilitates feedforward connections across distinct brain regions [34] and underpins the large-scale network activity essential for cognitive processing. Gamma rhythms have been implicated in diverse functions, including attention, long-term memory, working memory, perceptual grouping, language processing, and motor tasks [35,36,37,38,39,40,41].

Gamma oscillations also play a role in temporal encoding, sensory feature binding into coherent percepts, and the storage and recall of information [42,43,44]. These rhythms exhibit multifunctionality, supporting fundamental processes that are critical for sensory integration and higher-order cognitive functions [45]. Their involvement spans various cortical and subcortical systems, underscoring their importance in maintaining the brain’s computational efficiency and coherence during complex tasks [9,45,46].

### 2.4. Theta Rhythm

The theta rhythm, first described by Jung and Kornmüller, refers to oscillations in the frequency range of 4.5–7 Hz. These oscillations originate primarily in the hippocampus, particularly in the CA1 layer, receiving major inputs from the entorhinal cortex (EC) either directly via the EC→CA1 pathway or indirectly via the CA3→CA1 projection [47]. Theta oscillations dominate the early stages of sleep, including rapid eye movement (REM) sleep, where they are most prominent in the hippocampus, but they are also recorded in cortical and subcortical structures due to their role in modulating cortical activity patterns [48,49,50].

During wakefulness, hippocampal theta oscillations play a key role in organizing place cell firing into sequences, reflecting a state of readiness to process incoming signals [51]. This organization contributes to several aspects of memory formation [52], including the separation of encoding and retrieval dynamics, the enhancement of sequence retrieval, the selection of novel information for episodic encoding in the entorhinal cortex, and an influence on memory-guided action selection [53]. The precise timing of hippocampal activity modulated by theta oscillations during wakefulness is critical for the encoding of spatial memory and its subsequent consolidation during non-REM (NREM) sleep.

Theta oscillations also interact with sharp-wave ripples during NREM sleep, facilitating the consolidation of spatial memories. During REM sleep, transient increases in theta frequency and power—referred to as phasic REM—enhance hippocampal firing rates and coordination with cortical areas [54,55]. This synchronization supports the finetuning of memory processes, including both consolidation and the integration of spatial and episodic information.

### 2.5. Delta Rhythm

The delta rhythm, first described by Grey Walter, refers to slow, high-amplitude oscillations in the frequency range of 0.5–4 Hz. Delta waves are typically associated with deep stages (3 and 4) of non-rapid eye movement (NREM) sleep and can also be detected as intermittent delta activity in specific cortical regions, including the frontal (FIRDA), temporal (TIRDA), and occipital (OIRDA) cortices. Delta oscillations primarily originate in the thalamus, where they are driven by coordinated activity with the reticular formation. They can also arise in the cortex. These oscillations play a critical role in regulating physiological processes, including the release of hormones, such as growth hormone, which is tightly linked to slow-wave sleep [56,57].

Brain global oscillation is an inherent property of balanced systems, with its frequency determined by the time constants of its constituents [58]. In the absence of disturbances, neuronal activity rhythms remain stable, following a deterministic oscillatory dynamic primarily originating from the complex thalamocortical system. However, disruption of these balanced system dynamics is a prerequisite for various cerebral functions. The most complex functions find their physiological substrate in dynamic oscillation patterns, which arise within local neuronal ensembles or across diffuse networks of neurons [59].

Despite our continuous interaction with the external world, the perception of stimuli is not a constant cerebral event. Instead, it is subject to dynamic changes in network processing [60].

Input selection and plasticity rely on transient oscillatory perturbations generated at both the single-neuron and network levels. The eigenfrequency of these oscillations depends on the high- or low-pass filtering properties of neurons. Input selection exploits frequency characteristics and the frequency-tuning preferences of cortical interneurons, which shape network dynamics. This process occurs in the context of rhythmic cortical feedback to the thalamus, where the increasing recruitment of specific thalamocortical oscillations reduces neocortical responsiveness to external stimuli [61].

Input selection is further refined by phase biasing, as oscillation-related fluctuations in neuronal membrane potentials create discrete temporal windows for neuronal responses. Inputs that are not appropriately timed are ignored or delayed, whereas afferent activity that is properly coordinated can be amplified. This coordination can also bias the magnitude and direction of spike-timing-dependent plasticity [62,63,64]. For example, in the hippocampus, brief pulse trains delivered at the peak of theta oscillations induce long-term potentiation, while those delivered out of phase weaken synaptic inputs [65]. Conversely, rhythmic inputs that are out of phase can suppress specific oscillations in target networks [66].

The integration of information requires synchrony among convergent inputs, defined as the temporal window during which traces of earlier events are retained and alter responses to subsequent events [11].

Information processing, transfer, and storage occur within distributed networks of neuronal groups that are transiently synchronized by dynamic connections. The ability to synchronize depends on the coupling strength and the alignment of natural oscillation frequencies [60]. Neurons with similar oscillation frequencies maintain synchrony even with weak synaptic links [67]. Gamma-frequency oscillations, in particular, connect neuronal assemblies distributed across widespread cortical regions, enabling the processing of various attributes of external stimuli. These oscillations last long enough to support elementary cognitive acts [46,68].

The consolidation and integration of information are based on waking experiences, which create spike-sequence patterns within the thalamocortical system. These patterns influence the brain’s unperturbed default state [69,70,71]. During sleep, synaptic modifications induced by learning are solidified within self-organized oscillatory networks, ultimately contributing to long-term memory formation through functional and structural synaptic changes.

Cognitive and memory performance are associated with specific oscillatory dynamics. A tonic increase in alpha power and a decrease in theta power are linked to improved performance, while a large phasic decrease in alpha power and an increase in theta power are observed during memory-related tasks. The extent of upper alpha desynchronization positively correlates with semantic long-term memory performance, whereas theta synchronization is linked to the encoding of new information. Theta oscillations in hippocampo–cortical feedback loops are critical for encoding, while search and retrieval processes in semantic long-term memory are reflected in upper alpha oscillations within thalamo–cortical feedback loops [14]. For a detailed overview, see Table 1, which outlines the roles of different oscillatory bands in memory and cognitive functions.

Notably, while attention-demanding cognitive tasks, such as sensory or memory encoding, increase the activation of specific brain regions associated with the task at hand, they also lead to widespread deactivation in the distributed brain regions involved in large-scale structurally or functionally connected networks. These activations and deactivations can be measured through relationships among electrical activity, cerebrovascular hemodynamics, oxygen consumption, and the metabolism of neurons and glia. Key networks include the frontoparietal network, which supports executive functions and working memory; the dorsal and ventral attention networks, essential for goal-directed and stimulus-driven attentional processes [92]; and the default mode network (DMN), encompassing the precuneus, hippocampus, and retrosplenial cortex. The DMN is profoundly active during inwardly oriented mental activities, such as autobiographical and episodic memory [93,94,95].

Neurodegenerative diseases, particularly proteinopathies, are characterized by the aberrant accumulation of misfolded proteins, leading to synaptic dysfunction and disruptions in the structural and functional connectivity of brain networks. Synaptopathy and its EEG correlates, optimized through Fourier transform analyses, serve as biomarkers that precede brain tissue loss and track the temporal progression of the disease [96]. In cognitive impairment, slower oscillations (theta and delta rhythms) dominate over faster oscillations (alpha and beta rhythms). The increasing slow-to-fast ratio correlates with disease progression and the decline in brain complexity (or entropy), which reflects the brain’s capacity for dynamic changes associated with learning, memory, and social behavior. In AD, alterations in cholinergic transmission are associated with reduced delta amplitude and decreased theta and alpha synchrony compared to healthy individuals [97].

**Figure 1:**
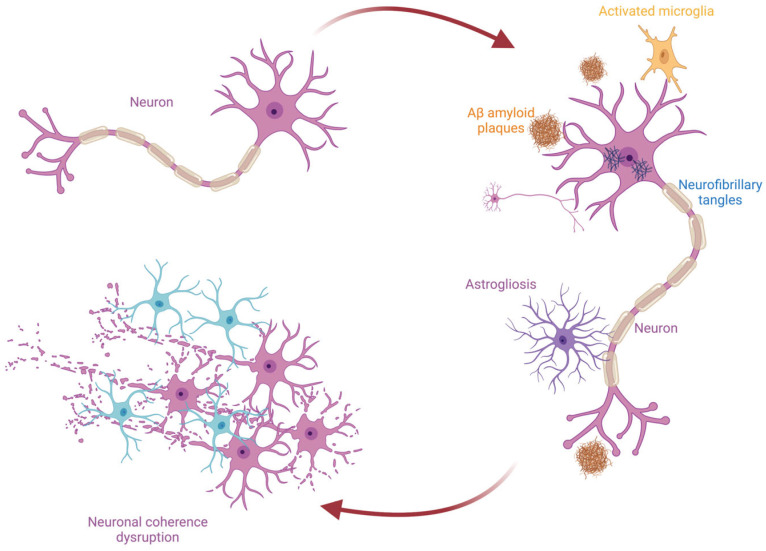
Proposed mechanism underlying neuronal dysfunction in Alzheimer’s disease leading to neuronal coherence disruption.

Synchrony plays a critical role in regulating the functional states of brain networks. Dysfunctions in synchrony, such as the abnormal activation or deactivation of task-relevant brain regions, contribute to cognitive impairment. For example, hippocampal hyperactivation and reduced DMN deactivation during memory encoding tasks have been observed in cognitively normal individuals at risk of AD. These include individuals with cerebral amyloid deposition [98], APOE ε4 carriers [99,100], familial AD mutation carriers [101,102], and those with mild cognitive impairment (MCI) [103,104]. While hippocampal hypoactivation during memory encoding and persisting DMN deactivation have been observed in the later stages of AD [101,105], the early pattern of hyperactivation may represent a compensatory mechanism in response to emerging cognitive decline, whereas later hypoactivation likely reflects network failure and system breakdown [106] (see Figure 1).

Alterations in the global coherence and global correlation dimensions, which measure connectivity across brain networks, are not uniformly affected across all frequency ranges. These differences may reflect the selective impact of pathological processes on the dynamics underlying different frequency bands [107]. Overall, the evidence suggests that network hyperactivity is an early biomarker in the pathogenesis of AD and represents a critical target for therapeutic intervention to prevent or reverse dysfunctions.

For an overview of the key findings on the dysregulation of neural oscillations in AD pathology, see Table 2 (mouse models) and Table 3 (human patients).

## 3. Brain Stimulation Techniques

A range of brain stimulation techniques has demonstrated potential for modulating neural oscillations to counteract cognitive dysfunctions in AD. These techniques include invasive brain stimulation (IBS), such as deep brain stimulation (DBS), and non-invasive brain stimulation (NIBS), encompassing such methods as repetitive transcranial magnetic stimulation (rTMS), transcranial direct current stimulation (tDCS), transcranial alternating current stimulation (tACS), transcranial temporal interference electrical stimulation (tTIS), sensory stimulation (visual and/or auditory), photobiomodulation (PBM), and transcranial ultrasound (transcranial-focused ultrasound (tFUS) and transcranial pulse stimulation (TPS)) [92].

### 3.1. Invasive Brain Stimulation

#### Deep Brain Stimulation

DBS involves the surgical implantation of electrodes into specific brain regions to deliver electrical stimulation. This approach modulates circuits involved in memory and cognition by promoting neurotransmitter release, enhancing neurogenesis, and stimulating the release of growth factors [142].

### 3.2. Non-Invasive Brain Stimulation

#### 3.2.1. Repetitive Transcranial Magnetic Stimulation

rTMS generates high-intensity magnetic fields that induce electrical currents in the brain, targeting specific neuronal populations. The effects of rTMS can propagate trans-synaptically across neural networks [92,143,144], modulating diminished cortical plasticity, restoring neural coherence, and stimulating the release of neurotransmitters, such as GABA, glutamate, dopamine, and acetylcholine [145,146]. Depending on the stimulation frequency, rTMS can enhance cortical excitability with high-frequency stimulation (>5 Hz) or reduce it with low-frequency stimulation (<1 Hz). This property is, therefore, useful for modulating diminished cortical plasticity and neural disconnection, restoring lost coherence, and stimulating cortical reactivity and neurotransmitter release (such as GABA, glutamate, dopamine, and acetylcholine), all of which play a role in AD pathogenesis [145,146]. A specific application of this technique, theta-burst stimulation (TBS), can be delivered using intermittent or continuous protocols to increase or decrease cortical excitability, respectively.

#### 3.2.2. Transcranial Direct Current Stimulation

tDCS employs a low-intensity continuous electric current (1–2 mA) to modulate neuronal resting membrane potentials involving changes in GABA and glutamate receptors [147]. This technique enhances or suppresses cortical excitability depending on the polarity of the stimulation, with anodal tDCS increasing excitability and cathodal tDCS decreasing it. Through its effects on synaptic reorganization, excitatory/inhibitory balance, and cortical connectivity, tDCS has shown potential for slowing disease progression and improving cognitive functions in AD [92].

#### 3.2.3. Transcranial Alternate Current Stimulation

tACS is a non-invasive neuromodulating technique which exploits the electrical fields generated by an alternating (sinusoidal) electric current (typically at 1–2 mA of intensity) via an anode and a cathode which alternate polarity at specific frequencies to modulate brain neural oscillations. This oscillating current can potentially entrain or disrupt brain rhythms according to the frequency and phase applied. By modulating neural oscillations, tACS improves intracortical connectivity and cholinergic transmission. Gamma-tACS, in particular, has shown efficacy in modulating gamma oscillations, whose disruption in AD correlates with disease severity, as well as theta oscillations, which are critical for memory and attention [9,148,149].

#### 3.2.4. Transcranial Temporal Interference Electrical Stimulation

tTIS is a non-invasive novel steerable deep stimulation technique which uses various kHz-range electric fields, thus allowing it to more accurately reach deep brain structures, such as the striatum and the hippocampus [150,151,152].

#### 3.2.5. Sensory Stimulation

Sensory stimulation induces brain oscillations through time-varying sensory inputs, such as visual (light), auditory (sound), and vibrotactile (tactile vibration) stimuli. These stimuli can be applied individually or in a multisensory manner.

#### 3.2.6. Photobiomodulation

PBM employs light in the red to near-infrared spectrum, delivered by lasers or light-emitting diodes, to target mitochondrial cytochrome-c oxidase. This process activates signaling molecules, such as cyclic AMP, enhances mitochondrial function, and boosts ATP production [153]. Preclinical studies indicate that PBM reduces amyloid plaques, neurofibrillary tangles, and oxidative stress markers, while promoting cellular health and synaptic function [153].

#### 3.2.7. Transcranial Ultrasound

Transcranial-focused ultrasound (tFUS) utilizes ultrasound frequencies below 700 kHz to penetrate deep brain regions, such as the hippocampus and medial temporal lobe, with high spatial specificity. This technique can enhance or suppress neuronal excitability, modulate short-term connectivity, and induce long-term plasticity. Additionally, tFUS may transiently alter blood–brain barrier permeability, allowing targeted delivery of therapeutic agents. In preclinical studies, tFUS has demonstrated amyloid reduction and behavioral improvements [154].

Transcranial pulse stimulation (TPS) utilizes single, ultrashort, repetitive ultrasound pulses to reach very deep brain structures, allowing doctors to adapt the stimulation parameters to better correspond to an individual targeted patient’s treatment. TPS induces neuroplasticity changes through metabolic, vascular, and neurotrophic modifies [155].

## 4. Preclinical Models: Insights into Modulating Neural Oscillations

Preclinical studies utilizing transgenic animal models of AD have provided invaluable insights into the potential of neural oscillation modulation for cognitive enhancement. These models are typically generated using pronuclear injection or gene-targeted replacement, incorporating specific mutations or isoforms relevant to AD pathology. The phenotypes exhibited by these models depend on the genetic modifications introduced and other determinants, such as the promoters driving transgene expression (e.g., PDGF-β, Thy-1, PrP, NSE, GFAP).

One prominent category of transgenic models focuses on amyloid precursor protein (APP) mutations. Examples include the PDAPP model, characterized by extracellular amyloid deposits, neuritic plaques, synaptic loss, astrocytosis, and cognitive impairments [156]. Tg2576 mice exhibit a fivefold increase in amyloid-beta 40 levels and a fourteenfold increase in amyloid-beta 42/43 levels, leading to widespread plaque formation in cortical and limbic structures and memory deficits [157]. Similarly, APP23 mice overexpress APP by sevenfold, resulting in senile plaques, gliosis, and dystrophic cholinergic fibers [158]. Other notable models include TASD-41 [159], J20 [160], and TgCRND8 [161], with each demonstrating unique pathological features, such as early amyloid deposition, synaptic dysfunction, and tau pathology.

Another important category includes the APP and presenilin 1 (PSEN1) models. The PSAPP model incorporates the APP_Swe_ and PS1_M146L_ mutations, leading to elevated amyloid-beta 42 levels and earlier amyloid deposition compared to Tg2576 mice [162]. The 5xFAD model combines mutations in APP_Swe_, APP_Lnd_, APP_Flo_, PS1_M146L_, and PS1_L286V_, resulting in high intracellular amyloid-beta 42 levels, extracellular amyloid deposits, synaptic dystrophy, neuronal loss, and cognitive decline [163]. These models allow for the study of accelerated pathology and its effects on neural networks and oscillatory dynamics.

APP models combined with apolipoprotein E (ApoE) mutations, such as TgCRND8× ApoEKI, demonstrate the interaction between amyloid pathology and ApoE4. This interaction results in increased IL-1β and GFAP reactivity, along with altered circadian rhythms [164]. Another model, APP23 × OM, introduces the APP_Swe_ mutation alongside leptin ob/ob, leading to hippocampal long-term potentiation deficits, tau cleavage, and hyperphosphorylation [165].

In animal models, DBS has demonstrated efficacy in targeting the fornix, entorhinal cortex, and nucleus basalis of Meynert (NBM). These interventions have proven effective in reducing amyloid burden, inflammation (e.g., decreasing astrogliosis and microglial activation), and neuronal loss [166]. Additionally, DBS has been shown to modulate CA1 theta–gamma oscillations, preserve memory [167], and exert neuroprotective effects [168].

NIBS has also yielded promising results in preclinical models. rTMS has been shown to restore functional synaptic connections through various mechanisms, such as long-term potentiation and ion channel activity [169]. It has also been found to enhance hippocampal neurotrophins, NMDA receptor content, and neurogenic proteins. Moreover, rTMS reduces neuronal excitability in the dentate gyrus, decreases amyloid-beta and phosphorylated tau deposits [170], and decreases pro-inflammatory cytokines, including IL-6 and TNF-α [171,172]. Notably, rTMS inhibits BACE1 expression and increases the ratio of insulin-degrading enzyme (IDE) [173,174], improving both the production and clearance of amyloid load by enhancing brain drainage pathways [174].

Although experimental evidence for tDCS in animal models remains limited, it has demonstrated the ability to decrease amyloid-beta plaque load and improve learning and memory [175,176]. Similarly, gamma-tACS has shown promise in enhancing synaptic transmission, which could potentially improve cognitive functions [177].

Sensory stimulation at gamma frequencies has also been shown to reduce pathological protein loads in AD mouse models, including amyloid-beta, phosphorylated tau, and altered microglia. This effect was first observed using GENUS (gamma entrainment using sensory stimuli), which employs light flickering and/or sound at 40 Hz to modulate brain activity. This approach targets multiple brain cell types, particularly fast-spiking parvalbumin-positive (FS-PV) interneurons. In 5xFAD mice, these interneurons reduce the levels of both Aβ40 and Aβ42 isoforms when entrained at 40 Hz. While the initial implementation of this method relied on optogenetic stimulation with optical fiber implants, its translational feasibility has improved significantly with the development of visual and auditory stimulation protocols at the same gamma frequency [178,179]. Sensory stimulation has also been shown to preserve cortical plasticity and improve cognitive performance [179].

PBM has demonstrated significant reductions in amyloid-beta plaques in the hippocampus and neocortex, with efficacy dependent on the administered dose and therapy protocol. PBM also enhances mitochondrial function, increases ATP production and c-Fos levels, and reduces hyperphosphorylated tau, neurofibrillary tangles, and oxidative stress markers [180,181].

tFUS has shown the ability to directly modulate the amyloid load in the hippocampus and cortex of 5xFAD mice. This technique utilizes 40 Hz ultrasound to reduce Aβ42 levels, offering a novel approach to addressing amyloid pathology [182].

While preclinical models have provided valuable insights into the mechanisms and potential benefits of neuromodulation in AD, their findings do not always translate directly to human clinical trials. Differences in brain structure, disease progression, and neurophysiological responses between animal models and patients with AD highlight the need for carefully designed clinical studies to confirm and refine these interventions for therapeutic use. Nevertheless, these results have encouraged researchers to pursue clinical studies involving patients with varying degrees of cognitive impairment.

## 5. Clinical Studies in Alzheimer’s Disease: Targeting Neural Oscillations

IBS and NIBS have demonstrated varying degrees of efficacy in improving brain plasticity, resulting in cognitive benefits and enhanced quality of life for AD patients [183].

DBS, with its ability to precisely target specific brain regions, is particularly applicable in AD treatment. The three primary target regions for DBS are the fornix, the NBM, and the ventral capsule/lateral striatum (VC/VS). The fornix, as a component of the Papez circuit, is crucial for memory formation and retrieval due to its direct connection with the hippocampus. DBS in the fornix has been associated with slower cognitive deterioration and higher cerebral glucose metabolism [184]. The NBM, a major producer of acetylcholine (deficient in AD), has demonstrated its role in stabilization of cognitive performance and improved cerebral glucose metabolism when targeted [185]. The VC/VS, part of the brain’s reward system, influences motivation and mood, and its stimulation has been linked to a slower rate of cognitive decline [186].

NIBS techniques, such as rTMS, have also been employed to target brain regions critical for cognition and AD pathology. Key regions include the dorsolateral prefrontal cortex (DLPFC), involved in working memory and executive functions [149,174,187,188,189,190,191,192,193,194,195,196,197,198,199,200,201,202,203,204,205,206,207,208,209,210,211]; the parietal cortex, relevant for spatial attention and episodic memory [190,196,203,204,208,210,212,213,214]; and the precuneus, a crucial node of the default mode network (DMN) and a region associated with episodic memory [149,215,216]. Experimental protocols often target additional areas, including Broca’s and Wernicke’s areas [190,192,196,203,204,208,210], the vertex [217], the superior temporal gyrus [217], the inferior frontal gyrus [217,218], the temporal cortex [209,212], the prefrontal cortex [219], the posterior parietal cortex [149], and the cerebellum [220]. Stimulation of the DLPFC has demonstrated improvements in action naming capabilities, cognitive performance, and working memory tasks, with these effects being particularly durable when combined with cognitive training [201,204]. Stimulation of the precuneus has yielded sustained cognitive benefits, especially in episodic memory tasks [215,216], while stimulation of the left parietal cortex has improved cognitive performance and functional connectivity within the DMN.

tDCS has predominantly targeted the DLPFC [221,222,223,224,225,226,227,228,229], the temporoparietal cortex [230,231], the frontal, parietal and temporal cortices [229,232,233,234,235,236,237], and Broca’s and Wernicke’s areas [229]. The effects of tDCS depend on polarity and intensity, which influence immediate and long-term outcomes [231,233]. Targeting the left DLPFC has mitigated neuropsychiatric symptoms [223], enhanced working memory [234], and improved cognitive performance, as well as instrumental activities of daily living [221,231,236]. Bilateral DLPFC stimulation, either cathodal or anodal, has been associated with better visual recognition memory performance [221,235,236]. Stimulation of the temporoparietal areas has led to improvements in global cognition [231], while simultaneous stimulation of the bilateral angular gyrus with rTMS and tDCS has improved neuropsychiatric symptoms, global cognition, and sleep quality [238].

Although tACS is still in its early stages, it has shown promise. Target regions for tACS include the left temporoparietal cortex [239], left or right temporal cortex [240], dorsolateral prefrontal cortex [241], precuneus [242,243], and areas individualized based on amyloid PET imaging [244]. Gamma-tACS has been found to improve memory performance and cholinergic transmission [9,149,150,242], with varying responses based on genetic factors, such as APOE status and disease stage [243]. Additionally, gamma-tACS enhances blood perfusion in amyloid-laden areas [244] and increases gamma power in EEG, while reducing tau load in the medial temporal cortex, without affecting amyloid-beta levels or microglial activation [240].

Gamma-sensory stimulation, utilizing visual, auditory, or combined modalities, has been shown to enhance functional connectivity in the DMN and visual networks [245,246,247]. It also reduces hippocampal and ventricular atrophy, although it does not significantly alter amyloid burden [248,249].

PBM has demonstrated potential in improving memory and executive functions in pilot studies [250], while tFUS has shown benefits in immediate and recognition memory, as well as metabolic activity enhancement in the hippocampus [251]. Additionally, TPS has been shown to induce measurable neurophysiological changes in power (frontal and occipital), coherence (frontal, occipital, and temporal), entropy (temporal and frontal), and cross-frequency coupling (parietal–frontal, parietal–temporal, frontal–temporal) between distant brain regions [155], thus improving functional connectivity within memory networks and global cognition [251]. A recent randomized clinical trial of TPS in patients with AD has shown that TPS can improve cognitive scores and ameliorate depressive symptoms by inducing upregulation of functional brain activation and connectivity [252]

Table 4 provides a comprehensive overview of studies employing DBS, while Table 5, Table 6, Table 7 and Table 8 provide an overview of NIBS in AD treatment.

**Figure 2:**
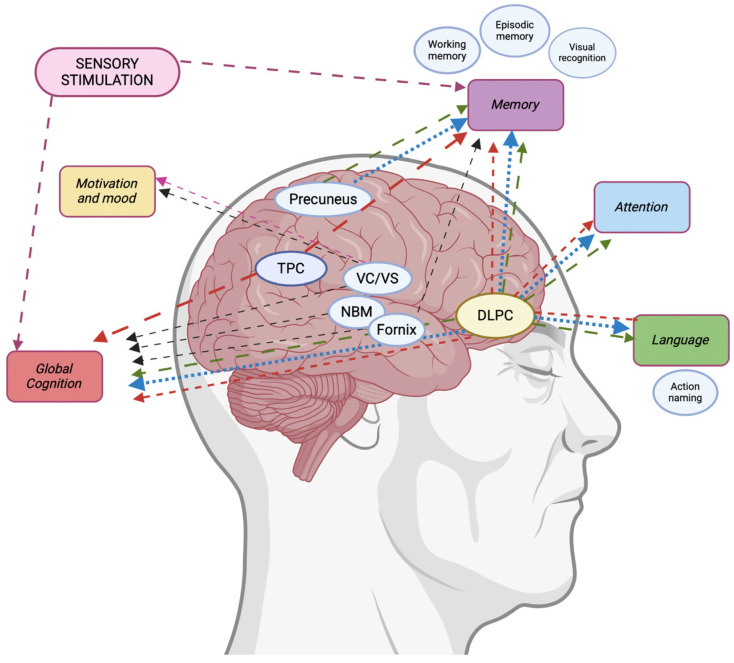
Summary of the evidence of IBS (DBS) and NIBS (rTMS, tDCS, tACS, sensory stimulation, and TPS) techniques in improving cognitive functions in AD patients. NIBS techniques based on magnetic or electrical stimulation (rTMS, tDCS, tACS) commonly target the dorsolateral prefrontal cortex (DLPFC), the precuneus, or the temporoparietal cortex (TPC), while NIBS techniques based on sensory or electrical stimulation (auditory/visual, tACS) target specific rhythms. IBS (DBS) and NIBS techniques based on ultrasound (TPS) target deep brain structures. Dashed arrows denote the degree of evidence for each technique (more dots = higher). Red arrows show tDCS, blue arrows show rTMS, green arrows show tACS, purple arrows show sensory stimulation, and black arrows show DBS. rTMS = repetitive transcranial magnetic stimulation; tDCS = trascranial direct current stimulation; tACS = alternate current stimulation; sensory stimulation = visual and/or auditory stimulation. VC/VS = ventral capsule/lateral striatum; NBM = nucleus basalis of Meynert.

All the neuromodulation techniques discussed have shown promising results in advancing our understanding of AD and its treatment, albeit with varying degrees of success and differing risk–benefit profiles. Figure 2 summarizes the different targets of different neuromodulatory interventions.

DBS offers the advantage of precise targeting of specific brain regions, particularly targeting the fornix, a region integral to memory circuitry. However, its invasive nature carries significant risks, including infections, hemorrhage, iatrogenic neurological disorders, and high costs. Furthermore, unresolved challenges include the standardization of intervention timing, stimulation parameters, and long-term efficacy and safety.

NIBS techniques, in contrast, are safer and less invasive while maintaining non-inferiority in effectiveness compared to DBS [273,274,275]. rTMS, tDCS, and tACS have particularly demonstrated potential in enhancing cognitive performance and delaying disease progression.

rTMS can modulate long-range connectivity and induce durable changes in neural plasticity, thanks to its capability of inducing depolarization or hyperpolarization of neurons [95]. tDCS leverages changes in membrane potentials to modulate neuronal excitability [276,277], while tACS and sensory stimulations effectively entrain neuronal oscillations [183,278]. Techniques, such as rTMS and tDCS, have demonstrated improvements in global cognition, memory, attention, and language, as well as reductions in neuropsychiatric symptoms. tACS modulates brain oscillations associated with memory deficits, while sensory stimulation has been shown to benefit both memory and global cognition.

Among NIBS techniques, rTMS is the most extensively studied, followed by tDCS, and then tACS, sensory stimulation, PBM, tFUS, and TPS. tDCS has shown high heterogeneity about the effectiveness of the results, suggesting that the intensity of stimulation could be a significant source of this variability. Despite these promising results, studies on the effects of tACS, sensory stimulations, and TPS in AD is still small, and larger, well-controlled trials are needed to confirm these preliminary findings.

While both DBS and NIBS have demonstrated efficacy in modulating neural activity in AD, they differ significantly in indications, benefits, and limitations. DBS provides precise targeting of deep brain structures, but is invasive, costly, and associated with surgical risks. In contrast, NIBS techniques, such as rTMS, tDCS, tACS, and TPS, offer non-invasive alternatives with fewer risks and greater accessibility, though their effects may be less durable and require repeated sessions. The choice between these modalities depends on disease stage, patient suitability, and treatment goals, highlighting the need for further research to optimize protocols and long-term efficacy.

However, critical challenges remain, including identifying optimal target populations, establishing standardized protocols (e.g., frequency, intensity, duration, and targeted regions), and fully understanding the underlying mechanisms. Additionally, long-term efficacy and safety are areas requiring further research [92,277]. Despite these challenges, positive outcomes have been reported for cognitive functions and markers of brain pathology. NIBS for AD has shown promising yet conflicting evidence, with level B evidence for rTMS and level C evidence for specific tDCS and tACS protocols [92,275,279]. Although no NIBS technique has been approved by the FDA for cognitive indications, a personalized rTMS system and a gamma-sensory stimulation device have recently received FDA breakthrough device designation for digital therapeutics in AD.

## 6. Challenges and Future Directions

While the concept of targeting neural oscillations in AD has gained traction, several critical challenges must be addressed to translate these insights into routine clinical practice. A central issue lies in the complexity and heterogeneity of oscillatory dysfunction. Changes in oscillation power, frequency, phase, and coherence vary not only between individuals but also within the same patient across different disease stages, cognitive domains, and contexts. For instance, some patients may exhibit pronounced alpha slowing and diminished gamma activity, while others might present atypical theta patterns or hyperactivation in specific networks. The identification of stable and reliable biomarkers that can guide individualized therapy is, therefore, paramount.

Technical and methodological complexities also persist. Non-invasive stimulation modalities, such as tACS and rTMS, must refine their targeting precision to reach deep brain structures or highly specific cortical regions reliably. While advanced coil designs, sophisticated head modeling, and integration with neuroimaging data (e.g., MRI-guided neuronavigation) are improving focality, ensuring that the applied stimulation resonates with the patient’s intrinsic oscillatory architecture remains challenging. Similarly, optimizing stimulation parameters—frequency, intensity, pulse shape, temporal patterning—and treatment schedules is still a trial-and-error process. The development of robust computational models which predict patient-specific responses to given stimulation parameters could streamline protocol refinement and reduce the time needed to identify optimal interventions.

Furthermore, there is a pressing need to embrace adaptive, closed-loop stimulation systems. Rather than using pre-set parameters, these systems would monitor the patient’s ongoing brain activity in real time and adjust stimulation delivery accordingly, capitalizing on critical windows for enhancing synaptic plasticity or interrupting pathological synchronization. Such closed-loop approaches may prevent over- or under-stimulation and lead to more durable cognitive improvements.

Complementary to these technological advances is the opportunity to integrate oscillation-targeted therapies with other intervention strategies. Pharmacological treatments which modulate neurotransmission in cholinergic, glutamatergic, or GABAergic systems could synergize with oscillation-based stimulations. Moreover, combining neuromodulatory approaches with cognitive rehabilitation, exercise programs, nutritional interventions, or sleep hygiene optimization might produce cumulative benefits. In fact, cognitive performance improves when physical interventions, such as IBS and/or NIBS, are synergistically combined with biochemical and behavioral strategies. Biochemical enhancers include not only pharmaceuticals but also specific nutrients (e.g., glucose, caffeine, flavonoids, folic acid, omega-3 fatty acids), natural remedies (e.g., herbal supplements), and even certain recreational drugs (e.g., nicotine) that have been investigated for their potential cognitive benefits. Additionally, pharmacological agents classified as nootropics, such as piracetam, modafinil, and ampakines, have been explored for their effects on attention, memory, and executive function, although their long-term efficacy and safety remain debated [280]. Behavioral strategies play a crucial role in cognitive enhancement and include good-quality sleep, regular physical exercise, structured cultural activities, and meditation training. Furthermore, neurofeedback, a technique that enables individuals to self-regulate brain activity through real-time EEG-based feedback, has demonstrated potential in enhancing cognitive performance and neural plasticity [281]. Tailoring these multimodal regimens to align with a patient’s genetic risk profile, biomarker status (e.g., amyloid or tau load), and clinical trajectory could improve both the efficacy and sustainability of outcomes.

Addressing logistical and ethical considerations is also essential. As emerging technologies move into the home environment, issues related to patient safety, device usability, data privacy, and long-term adherence must be carefully managed. Streamlined designs, telemonitoring capabilities, and user-friendly interfaces will be crucial for ensuring which patients and caregivers can comfortably incorporate these treatments into daily life. Meanwhile, large-scale, multi-center trials are needed to confirm efficacy across diverse populations and disease stages, and to refine stratification criteria which identify those most likely to benefit.

Ultimately, advancing this field hinges on interdisciplinary collaborations. By uniting neuroscientists, clinicians, biomedical engineers, computational modelers, and regulatory bodies, we can accelerate the development of best practices, foster method standardization, and promote data sharing and meta-analyses. Overcoming these challenges will pave the way for more precise, responsive, and ultimately more effective oscillation-based therapies in AD.

## 7. Limitations

This review provides a comprehensive synthesis of neuromodulation and neural oscillations in AD, but some limitations must be acknowledged. First, as a narrative review, study selection was based on relevance rather than predefined inclusion/exclusion criteria, posing a risk of selection bias. Second, the studies reviewed exhibit high heterogeneity in design, sample size, stimulation protocols, and cognitive assessments, making direct comparisons challenging. Standardization of the neuromodulation parameters and patient stratification is needed to improve reproducibility. Third, while preclinical models offer valuable mechanistic insights, their translational applicability to humans remains uncertain, and the long-term efficacy and safety of NIBS in AD are still unclear. Lastly, emerging techniques, such as transcranial pulse stimulation and transcranial-focused ultrasound, require further clinical validation through large-scale trials. Despite these limitations, this review highlights key advancements and challenges in the field, guiding future research directions.

## 8. Conclusions

The therapeutic targeting of neural oscillations represents an exciting new dimension in AD research and treatment. Early-stage studies have illuminated the roles of specific frequency bands—theta, alpha, beta, gamma—in memory formation, attention, and network integration. They have also demonstrated which external modulations of these rhythms, via techniques, like tACS or carefully placed DBS electrodes, can influence cognition, offering a tantalizing glimpse of what may be possible as methods mature.

However, the journey from proof-of-concept to clinically meaningful intervention is still underway. There is much to learn about the precise mechanistic underpinnings of oscillatory dysfunction in AD, how these patterns evolve as pathology advances, and how to restore or retune them most effectively. The promising direction forward involves merging theoretical knowledge with practical innovation. Through personalized medicine approaches, guided by robust biomarkers and supported by computational modeling, clinicians could one day prescribe stimulation parameters as precisely as medications. Closed-loop neuromodulation, informed by real-time electrophysiological data, may allow for dynamic and context-sensitive finetuning of brain states which support cognition and memory.

Moreover, harnessing oscillations should not be viewed in isolation. Integrating neuromodulation with pharmacological agents which restore neurotransmitter balance, lifestyle interventions which support neural health, and cognitive training exercises which reinforce learning, can create synergistic effects. Despite promising advancements, challenges remain regarding the clinical accessibility and cost-effectiveness of these interventions. While DBS offers precise targeting of deep brain structures, its invasiveness and high costs limit widespread application. In contrast, non-invasive techniques, such as rTMS, tDCS, and tACS, are more affordable and accessible but require repeated sessions for sustained benefits. Future research should focus on optimizing stimulation parameters and exploring home-based or wearable neuromodulation technologies to improve scalability and long-term usability. This holistic approach aims not merely to slow cognitive decline but potentially to enhance residual cognitive function, contributing to a better quality of life for patients and easing the burdens on caregivers and healthcare systems.

In summary, while the field is still evolving, the conceptual and technological foundations for targeting neural oscillations in AD are strong. Continued interdisciplinary collaboration, rigorous methodological refinement, and thoughtful integration of multiple therapeutic modalities can usher in a new era of interventions. As these therapies advance and become more accessible, we move closer to the goal of modulating brain rhythms to preserve and enhance cognitive function, ultimately offering renewed hope in the struggle against Alzheimer’s disease.

## Figures and Tables

**Table 1:** Role of different oscillatory bands in memory and cognitive functions.

Cognitive Functions	Study	Involved Oscillation
Episodic memory encoding and episodic memory retrieval processes	Klimesch et al., 1997 [72] Klimesch et al., 1994 [73] Klimesch et al., 2007 [74]	Increase in theta power, desynchronization in the lower and upper alpha band.
	Sauseng et al., 2005 [75]	Increase in theta oscillations spread from anterior to posterior recording sites; when information is retrieved, theta oscillations spread to posterior from anterior sites.
	Jones et al., 2005 [76] Sirota et al., 2008 [77]	Increase in theta rhythms guides hippocampal–prefrontal interactions in encoding information.
	Colgin and Moser, 2010 [78]	Memory encoding and retrieval are coordinated by different frequencies of hippocampal gamma oscillations; transitions between slow and fast gamma may occur.
	Duzel et al., 2010 [79]	Temporal coordination of neocortical gamma oscillators by hippocampal theta oscillations is a mechanism by which information contained in spatially widespread neocortical assemblies can be synchronously transferred to the associative networks of the hippocampus.
	Fell et al., 2011 [59]	Increased phase synchronization has been observed during various memory processes, including long-term memory encoding and retrieval.
	Griffiths et al., 2019 [41]	Decreases in neocortical alpha/beta power reliably precede and predict increases in hippocampal “fast” gamma power during episodic memory formation; during episodic memory retrieval, however, increases in hippocampal “slow” gamma power reliably precede and predict later decreases in neocortical alpha/beta power.
Declarative long-term memory formation	Fell et al., 2002 [80]	Initial increase in rhinal–hippocampal phase synchronization followed by a later desynchronization in the gamma band.
Long-term memory formation in perceptual learning task	Gruber et al., 2002 [81]	Increase in spectral gamma power at parietal electrode sites for identified pictures; highly synchronization in the gamma band between posterior electrodes.
Semantic memory processes	Klimesch et al., 1994 [73]	Upper alpha band.
Working memory process	Stam et al., 2000 [82]	Lower alpha band dimension increase, alpha1 band desynchronization.
	Onton et al., 2005 [83]	In female subjects, higher dimension in the theta band, more desynchronization in the theta and alpha1 band.
	Gevins et al., 1997 [84]	Increase in frontal midline theta oscillations.
	Lee et al., 2005 [85]	Increase in frontal midline theta oscillations, decrease in slow, parietocentral alpha signal.
	Van Vugh et al., 2010 [40]	Increase in theta and alpha signals with performance improvement, increases in theta signals with both increased task difficulty and with practice, decrease in alpha signals in the difficult tasks, and increase in theta oscillations for structuring the recurrent interaction between neurons in different brain regions during working memory.
	Fell et al., 2011 [59]	Increase in gamma oscillations primarily in the hippocampus and medial temporal lobe in the maintenance of two classes of stimuli (both letters and faces) in working memory; increased phase synchronization has been observed during various memory processes, including working memory maintenance.
Recognition of faces and facial expressions	Basar, 2006 [86]	Superposition of delta, theta, alpha, beta, and gamma oscillations, as parallel activations of neural assemblies in different cortical locations; known and anonymous faces can be differentiated by means of oscillatory brain dynamics; the differentiation of facial expression induces significant change in alpha and theta oscillation.
Memory consolidation	Buzsaki, 2015 [71]	Hippocampal sharp-wave ripples assist in transferring compressed hippocampal representation to distributed circuits to support memory consolidation.
Memory processing	Basar et al., 2001 [87]	Increase in theta oscillations.
Memory maintenance	Jensen and Tesche, 2002 [88] Klimesch et al., 2007 [74]	Increase in theta oscillations in frontal brain regions.
Working memory maintenance	Miller et al., 2018 [89]	Interactions between different rhythms in distinct cortical layers: executive control via interplay between network oscillations in gamma in superficial cortical layers (layers 2 and 3), and alpha and beta in deep cortical layers (layers 5 and 6); deep-layer alpha and beta are associated with top-down information and inhibition; this regulates the flow of bottom-up sensory information associated with superficial layer gamma.
Retrieval of spatial memory	Bieri et al., 2014 [90]	Slow and fast gamma rhythms coordinate prospecting and retrospective spatial coding modes in hippocampal place cells, respectively.
Replay of previously stored memories	Carr et al., 2012 [91]	Transient increases in slow gamma power and synchrony across dorsal CA3 and CA1 networks of both hemispheres, during sharp-wave ripples in both awake and quiescent states.

CA3, CA1: cornu ammonis regions 3 and 1 of the hippocampus; LTM: long-term memory; SWR: sharp-wave ripples.

**Table 2:** Dysregulation of neural oscillations in AD animal models.

Study	Experimental Design	Outcome
Driver et al., 2007 [108]	Recording of the gamma-frequency activity evoked with bath application of 200 nm kainate in hippocampal slices from mice overexpressing the human amyloid precursor protein (APP_SWE_) mutation (TAS10).	Decrease in hippocampal gamma-frequency oscillations in young TAS10 mice versus wild-type littermates.
Villette et al., 2010 [109]	Bilateral corticohippocampal local field potential signals collections during behavioral sessions.	Decreased rhythmic GABAergic septal activity and memory-associated theta oscillations after hippocampal amyloid-β pathology in the rat.
Verret et al., 2012 [110]	Electroencephalographic recordings in hAPP mice.	Aberrant gamma oscillatory activity and cognitive disfunction due to dysfunctions in the parvalbumin cells and inhibitory synaptic activity in transgenic mice.
Pena-Ortega et al., 2012 [111]	Recordings of faster oscillations in Alzheimer’s disease-transgenic mice.	Decrease in entorhinal cortex beta–gamma bursts induced by amyloid beta is blocked involving GSK-3.
Goutagny et al., 2013 [112]	Electrophysiological field potential recordings in the hippocampus of young transgenic CRND8 mice.	A significant proportion of 1-month-old TgCRND8 mice showed robust alterations of theta–gamma cross-frequency coupling in the principal output region of the subiculum, before Aβ overproduction.
Klein et al., 2016 [113]	In vitro gamma oscillations in the medial (MEC) and lateral (LEC) entorhinal cortex of the transgenic amyloid precursor protein (APP)-presenilin 1 (PS1) mouse model of Alzheimer’s disease (AD) at 4–5 months of age.	Changes in rodents’ gamma oscillations in the entorhinal cortex at an early stage of AD.
Gillespie et al., 2016 [114]	Local field potential signals recording of hippocampal network activity in apoE3-KI and apoE4-KI mice.	Decrease in gamma power during hippocampal sharp-wave ripples in APOE4-KI mice.
Mably et al., 2017 [115]	Recording spikes from place cells in hippocampal subfield CA1, together with corresponding rhythmic activity in local field potentials, in the 3xTg AD mouse model.	Decreased gamma activity in mice models of AD.
Etter et al., 2019 [116]	J20-amyloid precursor protein AD mouse model.	Reduced slow gamma amplitude and phase-amplitude coupling to theta oscillations phase, displaying spatial memory loss.

APP: amyloid precursor protein; GSK-3: glycogen synthase kinase-3; hAPP: human amyloid precursor protein; MEC: medial entorhinal cortex; LEC: lateral entorhinal cortex; AD: Alzheimer’s disease; APOE4-KI: apolipoprotein E4 knock-in; TgCRND8: transgenic CRND8 mouse model; Aβ: amyloid-β.

**Table 3:** Dysregulation of neural oscillations in individuals with AD.

Study	Methodology	Study Population	Outcome
Alpha oscillations			
Leuchter et al., 1987 [117]	EEG, resting state	AD vs. multi-infarct dementia vs. controls	Decrease in EEG alpha coherence
Petit et al., 1993 [118]	EEG, resting state and REM sleep	AD vs. controls	Decrease in EEG alpha oscillations
Besthorn et al., 1994 [119]	EEG, resting state	50 AD vs. 42 controls	Decrease in EEG alpha coherence
Dunkin et al., 1994, [120]	EEG, resting state	AD patients	Decrease in EEG alpha coherence
Cook and Leuchter, 1996 [121]	EEG, resting state	AD patients	Decrease in EEG alpha synchrony
Chiaramonti et al., 1997 [122]	Quantitative EEG, resting state	31 AD	Decrease in EEG alpha oscillations
Jelic et al., 1998 [123]	EEG, resting state	14 AD	Decrease alpha/theta oscillations ratio
Locatelli et al., 1998 [124]	EEG, resting state	10 AD vs. 10 controls	Decrease in EEG alpha synchrony
Rodriguez et al., 1999 [125]	Quantitative EEG, resting state	48 AD	Decrease in EEG alpha oscillations
Huang et al., 2000 [126]	EEG, resting state	38 mild AD vs. 31 MCI	Decrease in alpha oscillations
Adler et al., 2003 [127]	EEG, resting state	31 AD vs. 17 controls	Decrease in EEG alpha coherence
Hogan et al., 2003 [128]	EEG, memory paradigm	10 AD vs. 10 controls	Decrease in alpha evoked coherence
Moretti et al., 2004 [129]	EEG, resting state	Mild AD patients	Decrease in alpha2 and alpha3 power
Zeng-Yan, 2005 [130]	EEG, photic stimulation	35 AD vs. 33 controls	Decrease in alpha evoked coherence
Babiloni et al., 2004 [131]	EEG, resting state and LORETA	48 mild AD vs. 20 vascular dementia vs. 30 controls	Decrease in alpha rhythms in central, parietal, temporal, and limbic
Babiloni et al., 2006 [132]	EEG, resting state	AD vs. controls	Decrease in alpha power
Guntekin et al., 2008 [133]	EEG, visual oddball	21 AD vs. 19 controls	Decrease in EEG alpha evoked coherence
Ponomareva et al., 2008 [134]	EEG, resting state	145 AD	Increased excitability and dysfunction of deep brain and alpha rhythm-generating structures
Beta oscillations			
Petit et al., 1993 [118]	EEG, resting state and REM sleep	AD vs. controls	Decrease in EEG beta oscillations
Besthorn et al., 1994 [119]	EEG, resting state	50 AD vs. 42 controls	Decrease in EEG beta coherence
Chiaramonti et al., 1997 [122]	Quantitative EEG, resting state	31 AD	Decrease in EEG beta oscillations
Wada et al., 1998 [135]	EEG, resting state and during photic stimulation	10 AD vs. 10 controls	Decrease in EEG beta synchrony
Rodriguez et al., 1999 [125]	Quantitative EEG, resting state	48 AD	Decrease in EEG beta oscillations
Huang et al., 2000 [126]	EEG, resting state	38 mild AD vs. 31 MCI	Decrease in beta oscillations
Zeng-Yan, 2005 [130]	EEG, photic stimulation		Decrease in EEG beta evoked coherence
Babiloni et al., 2006 [132]	EEG, resting state	AD vs. controls	Decrease in beta power
Delta oscillations			
Petit et al., 1993 [118]	EEG, resting state and REM sleep	AD vs. controls	Increase in EEG delta oscillations
Besthorn et al., 1994 [119]	EEG, resting state	50 AD vs. 42 controls	Increase in EEG delta oscillations
Chiaramonti et al., 1997 [122]	Quantitative EEG, resting state	31 AD	Increase in EEG delta oscillations
Wada et al., 1998 [135]	EEG, resting state and during photic stimulation	10 AD vs. 10 controls	Decrease in EEG delta synchrony
Rodriguez et al., 1999 [125]	Quantitative EEG, resting state	48 AD	Increase in EEG delta oscillations
Huang et al., 2000 [126]	EEG, resting state	38 mild AD vs. 31 MCI vs. 24 controls	Increase in delta oscillations
Moretti et al., 2004 [129]	EEG, resting state	AD patients	Increase in delta power
Babiloni et al., 2004 [131]	EEG, resting state and LORETA	AD patients	Increase in delta oscillations in occipital, temporal, and limbic brain regions
Babiloni et al., 2006 [132]	EEG, resting	AD vs. controls	Increase in delta power
Yener et al., 2007 [136]	EEG, Visual oddball	22 probable AD vs. 20 controls	Increase in delta oscillations
Yener et al., 2008 [137]	EEG, Visual oddball	17 MCI vs. 17 controls	Decrease in delta amplitudes
Guntekin et al., 2008 [133]	EEG, Visual oddball	21 AD vs. 19 controls	Decrease in delta evoked coherence
Theta oscillations			
Petit et al., 1993 [118]	EEG, resting state and REM sleep	AD vs. controls	Increase in EEG theta oscillations
Besthorn et al., 1994 [119]	EEG, resting state	50 AD vs. 42 controls	Increase in EEG theta oscillations
Chiaramonti et al., 1997 [122]	Quantitative EEG, resting state	31 AD	Increase in EEG theta oscillations
Jelic et al., 1998 [123]	EEG, resting state	14 AD	Decrease alpha/theta oscillations ratio
Wada et al., 1998 [135]	EEG, resting state and during photic stimulation	10 AD vs. 10 controls	Decrease in EEG theta synchrony
Rodriguez et al., 1999 [125]	Quantitative EEG, resting state	in 48 AD	Increase in EEG theta oscillations
Huang et al., 2000 [126]	EEG, resting state	38 mild AD vs. 31 MCI vs. 24 controls	Increase in theta oscillations
Yener et al., 2007 [136]	EEG, visual oddball	22 probable AD vs. 20 controls	Decrease in theta synchrony
Guntekin et al., 2008 [133]	EEG, visual oddball	21 AD vs. 19 controls	Decrease in theta evoked coherence
Babiloni et al., 2004 [131]	EEG, resting state	AD patients	No changes in theta oscillations
Babiloni et al., 2006 [132]	EEG, resting state	AD vs. controls	Increase in theta power
Prichep et al., 2006 [138]	EEG, resting state	Prodromic AD	Increase in theta power
Gamma oscillations			
Osipova et al., 2006 [139]	Whole-head magnetoencephalography	AD patients	Increase in magnetic auditory 40 Hz steady-state response
Van Deursen et al., 2008 [140]	EEG, resting state, music listening, story listening, and visual stimulation	15 AD vs. 20 MCI vs. 20 controls	Increase in EEG gamma oscillations
Van Deursen et al., 2011 [141]	Whole-head magnetoencephalography	15 AD vs. 20 MCI vs. 20 controls	Increase in 40 Hz steady-state response

EEG: electroencephalography; REM: rapid eye movement; AD: Alzheimer’s disease; MCI: mild cognitive impairment; LORETA: low-resolution brain electromagnetic tomography.

**Table 4:** Studies employing DBS in the treatment of individuals with AD.

Study	Target Area	Disease Stage	Tools and Scales	Main Outcomes
Turnbull et al., 1985 [253]	NBM	Mild–moderate AD	Global cognition, EEG, FDG-PET	No changes in clinical outcomes. Preservation of glucose metabolic activity in ipsilateral temporal and parietal lobes
Laxton et al., 2010 [254]	Fornix	Mild AD	MMSE, ADAS-cog, FDG-PET	Slower decline in cognitive scores. Increased glucose metabolism in temporal and parietal lobes
Fontaine et al., 2013 [255]	Fornix	Mild AD	MMSE, ADAS-cog, FDG-PET, FCSRT	Stabilization in cognitive scores. Increased glucose metabolism in mesial temporal lobes
Sankar et al., 2015 [256]	Fornix	Mild AD	MMSE, ADAS-cog, MRI	Slower hippocampal atrophy progression
Kuhn et al., 2015 [257]	NBM	Mild–moderate AD	ADAS-cog, EEG, FDG-PET	Stabilization in cognitive scores and improved glucose metabolism
Kuhn et al., 2015 [258]	NBM	Mild AD	MMSE, ADAS-cog	Stabilization in cognitive scores
Hardenacke et al., 2016 [259]	NBM	Mild AD	ADAS-cog	Slower decline in cognitive scores
Lozano et al., 2016 [260]	Fornix	Mild AD	ADAS-cog, CDR-SB, MRI, FDG-PET	Slower decline in cognitive scores. Increased glucose metabolism
McMullen et al., 2016 [261]	Fornix	AD	ADAS-cog, MRI, FDG-PET	Slower decline in cognitive scores. Increased glucose metabolism
Ponce et al., 2016 [262]	Fornix	AD	Safety	Safety of procedures
Durschmid et al., 2017 [263]	NBM	AD	EEG	Occurrence of early complex of EEG components under stimulation
Baldermann et al., 2018 [264]	NBM	AD	MMSE, ADAS-cog, ADAS-mem, MRI	Less advanced atrophy may profit from DBS of the NBM
Leoutsakos et al., 2018 [265]	Fornix	Mild AD	ADAS-cog, CDR-SB, CVLT, NPI	Safety of procedures
Scharre et al., 2018 [266]	VC/VS	AD with biomarkers	CDR, FDG-PET	Slower cognitive decline
Mao et al., 2018 [267]	Fornix	Severe AD	MMSE, MoCA, CDR	Safety and tolerability of procedures. Slower cognitive decline

NBM: nucleus basalis of Meynert; AD: Alzheimer’s disease; MMSE: mini-mental state examination; ADAS-cog: Alzheimer’s Disease Assessment Scale—Cognitive Subscale; FDG-PET: fluorodeoxyglucose positron-emission tomography; MRI: magnetic resonance imaging; FCSRT: free and cued selective reminding test; CDR: clinical dementia rating; CDR-SB: clinical dementia rating sum of boxes; ADAS-mem: Alzheimer’s Disease Assessment Scale—Memory Subscale; CVLT: California Verbal Learning Test; NPI: neuropsychiatric inventory; VC/VS: ventral capsule/ventral striatum; MoCA: Montreal Cognitive Assessment; EEG: electroencephalography.

**Table 5:** Studies employing rTMS in the treatment of individuals with AD.

Study	Target Area	Disease Stage	Tools and Scales	Main Outcomes
Cotelli et al., 2006 [188]	L/R DLPFC	Mild–moderate AD	Naming, MMSE	Significant enhancement in action naming capabilities
Cotelli et al., 2008 [189]	L/R DLPFC	Mild/moderate–severe AD	Naming, MMSE	Significant enhancement in action naming capabilities
Cotelli et al., 2011 [187]	L DLPFC	AD	BADA, MMSE	Enhancement in cognitive performance. Durability of improvement.
Bentwich et al., 2012 [190]	L/R DLPFC, Bro, Wer, L/R PC	Mild AD	ADAS-cog, CGIC	Enhancement in cognitive performance
Ahmed et al., 2012 [191]	L/R DLPFC	AD	MMSE, IADL, GDS	Enhancement in cognitive performance
Rabey et al., 2013 [192]	L/R DLPFC, Bro, Wer	Mild–moderate AD	ADAS-cog, CGIC	Enhancement in cognitive performance and neuropsychiatric symptoms
Eliasova et al., 2014 [218]	R IFG	MCI and mild AD	Stroop, TMT, CVSET	Significant improvement in attention and psychomotor speed
Devi et al., 2014 [193]	L/R DLPFC	AD	BDAE, MRI	Improvement in language domain
Rutherford et al., 2015 [194]	L/R DLPFC	AD	ADAS-cog, MoCA, RMBC	Enhancement in cognitive performance and slower cognitive decline
Wu et al., 2015 [195]	L DLPFC	AD	BEHAVE-AD	Combined medical and rTMS treatment improve both cognitive functioning and the behavioral and psychological symptoms
Anderkova et al., 2015 [217]	R IFG, R STG, VTX,		TMT, Stroop, MRI	Enhancement in cognitive performance. Distinct pattern of GM atrophy in MCI/AD diminishes the cognitive effects induced by rTMS of the temporal neocortex
Lee et al., 2016 [196]	Bro, Wer, L/R DLPFC, L/R PC	MCI and mild AD	ADAS-cog, MMSE, CGIC	Efficacy of combined therapy. Improvement in memory and language domains
Zhao et al., 2017 [212]	PC and TC	MCI and mild AD	ADAS-cog, MMSE, MoCA, AVLT	Efficacy of combined therapy. Improvement in cognitive function, memory and language
Koch et al., 2018 [215]	Precuneus	Prodromal AD with positive biomarkers	ADCS-PACC, EEG, TMS/EEG	Significant improvements in episodic memory tasks
Alcalà-Lozano et al., 2018 [197]	L DLPFC	AD	ADAS-cog, MMSE, NPI, GDS, CGIC	Improving cognitive function, behavior and functionality after 3 weeks of treatment, and the effects were maintained for 4 weeks more without treatment
Zhang et al., 2019 [198]	L DLPFC	AD	ADAS-cog, MMSE, ACE-III, NPI, MRI	Possible effects of rTMS-CT on preventing clinical and neuronal functional deterioration in the left DLPFC of AD patients
Turriziani et al., 2019 [199]	L/R DLPFC	AD	Memory, MMSE, RAVLT, other	Inhibitory rTMS over the R DLPFC can improve recognition memory function in AD patients
Padala et al., 2020 [200]	L DLPFC	AD	AES-C, MMSE, IADL, CGIC	Safety of stimulation. Improving apathy, function, and cognition
Bagattini et al., 2020 [201]	L DLPFC	MCI and mild–moderate AD	FNAT, MMSE, RAVLT, TMT, other	Significant improvements in cognitive performance
Wu et al., 2020 [202]	L DLPFC	Mild–moderate AD	MMSE, MoCA, GDS, NPI, MRI	Significant improvements in cognitive performance and neuropsychiatric symptoms.
Brem et al., 2020 [203]	L/R DLPFC, Bro, Wer, L/R PC	Mild–moderate AD	ADAS-cog, CGIC, TMS	Combined rTMS and cognitive training may improve the cognitive status of AD patients, with TMS-induced cortical plasticity at baseline serving as predictor of therapeutic outcome
Sabbagh et al., 2020 [204]	L/R DLPFC, Bro, Wer, L/R PC, L PC	Mild–moderate AD	ADAS-cog, CGIC	Combined therapy led to substantial improvements in cognitive functions and quality of life
Jia et al., 2021 [213]	L PC	Mild–moderate AD	MMSE, CDR, PVLT	Improvement in cognition scores
Li et al., 2021 [174]	L DLPFC	AD	ADAS-cog, MMSE	Improvement in cognition scores after 6 weeks of rTMS. The cortical plasticity improvement correlated to the observed cognition change
Liu et al., 2021 [268]	L/R angular gyrus	AD patients and 41 healthy controls	Neuropsychological assessments, MRI, EEG	40-Hz rTMS modulated gamma-band oscillations in the L posterior TP region. rTMS prevented GM volume loss, enhanced local functional integration within L/R angular gyrus, global functional integration in L/R angular gyrus and the L middle frontal gyrus, strengthened information flow from the L posterior TP region to the F areas, and strengthened the dynamic connectivity between anterior and posterior brain regions
Leocani et al., 2021 [219]	L/R PFC	AD	ADAS-cog, MMSE, BDI	Safety of stimulation. Beneficial, but transient, effects on cognition
Zhou et al., 2021 [205]	L/R DLPFC	Mild–moderate AD	PSQI, ADAS-cog	Improvement in cognition scores, but not in activities of daily living (ADL)
Teti Mayer et al., 2021 [206]	L DLPFC	Mild–moderate AD	MMSE, MDRS, QoL, other	Enhancement in semantic memory and reducing in anxiety.
Wu et al., 2022 [207]	L DLPFC	Mild–moderate AD	AM, MMSE, MoCA, CDR, NPI, other	Accelerated intermittent theta-burst stimulation of the DLPFC demonstrated an effective and well-tolerated complementary treatment for patients with AD
Vecchio et al., 2022 [208]	L/R DLPFC, Bro, Wer, L/R PC	Mild–moderate AD	ADAS-cog, EEG	Improvement in cognitive scales. Delta and alpha1 Small Word graph is a diagnostic biomarkers of AD, whereas the alpha2 Small Word graph represents a prognostic biomarker of cognitive recovery
Qin et al., 2022 [209]	L DLPFC, L TC	Mild–moderate AD	MRI, ADAS-cog, NPI, ACE-III, other	rTMS combined with cognitive training induced increased low frequency fluctuation neural oscillations and functional connectivity in brain regions subserving cognition
Suarez et al., 2022 [210]	L/R DLPFC, Bro, Wer, L/R PC	AD	ADAS-cog, MMSE, apathy	Combined treatment produced long-term improvement in apathy and more general cognitive improvement only in patients who responded well to the initial 6-week protocol
Yao et al., 2022 [220]	Cerebellum	AD	ADAS-cog, MMSE, MoCA, MRI, other	5 Hz rTMS of the bilateral cerebellum improves cognitive performance and brain connectivity modulation
Mimeza-Alvarado et al., 2022 [211]	L DLPFC	MCI and mild AD	ADAS-cog, FAB, VF, ADL, GDS,	Safety of application of fast gamma magnetic stimulation on L DLPFC twice a day for 6 months from home
Koch et al., 2022 [216]	Precuneus	Mild–moderate AD with biomarkers	CDR-SB, ADAS-cog, TMS/EEG, other	The group receiving precuneus rTMS maintained the performance, while the sham group displayed a decline
Wei et al., 2022 [214]	L PC	Mild–moderate AD	MMSE, PVLT, MRI	Increase in cognitive performance and dynamic functional connectivity in DMN
Casula et al., 2022 [149]	L DLPFC, Precuneus, L PPC	Mild–moderate AD	TMS-EEG, cognitive evaluation	Novel evidence that frontal lobe gamma activity is dampened in AD patients, which is measurable by TMS EEG

L/R DLPFC: left/right dorsolateral prefrontal cortex; Bro: Broca’s area; Wer: Wernicke’s area; PC: parietal cortex; PPC: posterior parietal cortex; TC: temporal cortex; PFC: prefrontal cortex; IFG: inferior frontal gyrus; STG: superior frontal gyrus; VTX: vertex; FAB: frontal assessment battery; VF: verbal fluency; AD: Alzheimer’s disease; MCI: mild cognitive impairment; MMSE: mini-mental state examination; ADAS-cog: Alzheimer’s Disease Assessment Scale—Cognitive Subscale; CGIC: clinical global impression of change; GDS: geriatric depression scale; BDI: Beck depression inventory; MRI: magnetic resonance imaging; TMS: transcranial magnetic stimulation; EEG: electroencephalography; CDR: clinical dementia rating; CDR-SB: clinical dementia rating sum of boxes; MoCA: Montreal cognitive assessment; RAVLT: Rey auditory verbal learning test; PVLT: phonemic verbal learning test; ACE-III: Addenbrooke’s cognitive examination-III; ADL: activities of daily living; QoL: quality of life; AM: autobiographical memory; MDRS: Mattis dementia rating scale; TMT: trail making test.

**Table 6:** Studies employing tDCS in the treatment of individuals with AD.

Study	Target Area	Disease Stage	Tools and Scales	Main Outcomes
Ferrucci et al., 2008 [234]	TC, PC	AD	Word recognition task	tDCS over the TP areas can affect recognition memory performance in AD patients
Boggio et al., 2009 [235]	TC	Mild–moderate AD	Stroop, Digit Span, VRM	Improvement on a visual recognition memory task after temporal and prefrontal tDCS
Boggio et al., 2012 [236]	TC	AD	ADAS-cog, MMSE, VRM, VAT	Anodal tDCS over the T cortex in 5 consecutive daily sessions improves visual recognition memory, and the improvement persists for at least 4 weeks after therapy
Cotelli et al., 2014 [221]	L DLPFC	AD	FNAT, MMSE, ADL, NPI, other	Anodal tDCS plus individualized computerized memory training improved performance after 2 weeks
Khedr et al., 2014 [222]	L DLPFC	AD	MMSE, IQ-WAIS, ERP, TMS	Repeated sessions of tDCS improve cognitive function and reduce the P300 latency
Suemoto et al., 2014 [223]	L DLPFC	AD	MMSE, apathy, NPI, ADAS-cog, other	Repeated anodal tDCS over the left DLPFC had no effect on apathy in elderly patients with moderate AD
Bystad et al., 2016 [237]	L TC	AD	CVLT, MMSE, TMT	Active tDCS stimulation did not significantly improve verbal memory function in AD
Roncero et al., 2017 [230]	Inferior TPC	AD	Memory	tDCS stimulation improves anomia
Khedr et al., 2019 [231]	L/R TPC	Mild–moderate AD	MMSE, CDT, MoCA, CDS	Improvement in the total score of each cognitive rating scale
Im et al., 2019 [224]	L DLPFC	Mild AD	MMSE, FDG-PET, other	Daily anodal tDCS over the DLPFC for 6 months improves or stabilizes cognition
Cespon et al., 2019 [225]	L DLPFC	AD	Reaction times, ERP	Appropriate tDCS parameters induce behavioral improvements
Inagawa et al., 2019 [226]	L DLPFC	Mild–moderate AD	ADAS-cog, MMSE, FAB	tDCS is safe and tolerable but causes no statistically significant cognitive effects
Liu et al., 2020 [232]	L/R FC, L/R TC	MCI and mild AD	ADAS-cog, MOCA	Improvements in specific memory tasks can be safely achieved after a single session of bitemporal tDCS
Rasmussen et al., 2021 [227]	L DLPFC	AD	MMSE, MRI	High-definition tDCS improves delayed memory in AD
Gangemi et al., 2021 [233]	L FC, L TC	Mild AD	MMSE, MODA	t-DCS intervention was effective both in the short- and the long-term to slow down the progression of AD on specific neurophysiological domains and on neurophysiological activity
Smirni et al., 2021 [228]	L/R DLPFC	Mild AD	VF	tDCS over DLPFC can improve verbal fluency tasks in AD patients
Andrade et al., 2022 [229]	L/R DLPFC, Bro, Wer, L/R PC	Mild–moderate AD	ADAS-cog, EEG	Anodal tDCS and cognitive stimulation improved cognitive function and changed EEG brain activity

TC: temporal cortex; PC: parietal cortex; L/R DLPFC: left/right dorsolateral prefrontal cortex; FC: frontal cortex; TPC: temporoparietal cortex; Bro: Broca’s area; Wer: Wernicke’s area; AD: Alzheimer’s disease; MCI: mild cognitive impairment; MMSE: mini-mental state examination; ADAS-cog: Alzheimer’s Disease Assessment Scale—Cognitive Subscale; MoCA: Montreal Cognitive Assessment; VAT: visual attention task; VRM: verbal recognition memory; FNAT: face–name association task; ADL: activities of daily living; NPI: neuropsychiatric inventory; IQ-WAIS: intelligence quotient from the Wechsler Adult Intelligence Scale; ERP: event-related potential; TMS: transcranial magnetic stimulation; CVLT: California Verbal Learning Test; TMT: trail making test; CDT: clock drawing test; CDS: cognitive decline scale; FDG-PET: fluorodeoxyglucose positron-emission tomography; FAB: frontal assessment battery; MODA: Milan Overall Dementia Assessment; VF: verbal fluency; EEG: electroencephalography; MRI: magnetic resonance imaging.

**Table 7:** Studies employing tACS in the treatment of individuals with AD.

Study	Target Area	Disease Stage	Tools and Scales	Main Outcomes
Kehler et al., 2020 [241]	L DLPFC	MCI or mild to moderate AD patients	WMS-IV and MADRS	tACS determines maintenance of cognitive improvement
Benussi et al., 2021 [242]	Precuneus	MCI with biomarkers	RAVLT, FNAT, TMS	Significant improvements in episodic memory and increased cholinergic transmission immediately after -tACS in AD patients
Bréchet et al., 2021 [239]	L TPC	AD with biomarkers	EEG, MoCA, memory index	Feasibility and safety of intervention
Sprugnoli et al., 2021 [244]	Individualized on amyloid PET	Mild–moderate AD	ASL-MRI, ADAS-cog, MMSE, EEG	Significant increase in blood perfusion in bilateral T lobes, correlating with improvements in episodic memory and changes in gamma band spectral power
Dhaynaut et al., 2022 [240]	L/R TC	Mild–moderate AD with biomarkers	Tau/amyloid/microglia-PET	Increase in gamma spectral power on EEG and a significant decrease in phosphorylated Tau burden following tACS treatment, primarily in the targeted left and right temporal lobe regions
Benussi et al., 2022 [243]	Precuneus	MCI and mild AD with biomarkers	RAVLT, FNAT, TMS, EEG, genetics	Significant improvements in episodic memory and increased cholinergic transmission immediately after tACS in early-stage AD

L DLPFC: left dorsolateral prefrontal cortex; L TCP: left temporoparietal cortex; L/R TC: left/right temporal cortex; MCI: mild cognitive impairment; AD: Alzheimer’s disease; WMS-IV: Wechsler Memory Scale—Fourth Edition; MADRS: Montgomery–Åsberg Depression Rating Scale; MoCA: Montreal Cognitive Assessment; RAVLT: Rey Auditory Verbal Learning Test; FNAT: face–name association task; TMS: transcranial magnetic stimulation; EEG: electroencephalography; PET: positron-emission tomography; ASL-MRI: arterial spin labeling magnetic resonance imaging.

**Table 8:** Studies employing sensory stimulation in the treatment of individuals with AD.

Study	Target Area	Disease Stage	Tools and Scales	Main Outcomes
Skjerve et al., 2004 [269]	BLT, illumination: 5000–8000 lux.	AD patients	CMAI, BEHAVE-AD, SWD, and wrist-worn actigraphy (Actiwatch).	Short-duration bright light improves behavioral symptoms and aspects of activity rhythm disturbances even in severe AD
Van Hoof et al., 2009 [270]	High-intensity light stimulation. Bluish (6500 K) and yellowish (2700 K) light	AD patients	GIP, tympanic temperature	Bright light reduces depressive symptoms and agitation
Burns et al., 2009 [271]	Full spectrum BLT (10,000 lux), standard fluorescent tube light (100 lux)	AD patients	MMSE, CSDD, CRBRS, MOUSEPAD, and CMAI	Bright light reduces agitation
Van Deursen et al., 2011 [141]	Auditory 40 Hz stimulation	AD patients	TRR, 40 Hz steady state response	40-Hz SSR might be a candidate marker of disease progression.
Clements-Cortes et al., 2016 [245]	Sound; vibrotactile- somatosensory (39.96–40.06 Hz)	From mild to moderate AD patients	SLUMS, OERS, and behavioral observation	40 Hz sound stimulation has a good impact on mild and moderate AD
Suk et al., 2020 [272]	Concurrent light and sound	AD patients	EEG, iEEG	Safety and feasibility of treatment. Sensory stimulation increased the spectral power and coherence at 40 Hz
He et al., 2021 [248]	Light and sound (40 Hz)	MCI due to AD patients	MRI, EEG, venous blood draws, lumbar punctures, and cognitive testing	Prolonged gamma sensory flicker is safe, tolerable, and feasible with preliminary indications of immune and network effects
Chan et al., 2021 [247]	Light and sound (40 Hz)	Mild AD patients	EEG, MRI, fMRI, actigraphy recordings, cognitive assessments	Safety and feasibility of treatment. Reduction in nighttime active periods. Improvement in functional abilities

BLT: bright-light therapy; AD: Alzheimer’s disease; CMAI: Cohen–Mansfield Agitation Inventory; BEHAVE-AD: Behavioral Pathology in Alzheimer’s Disease Rating Scale; SWD: sleep–wake disturbances; GIP: Groningen Interaction Protocol; MMSE: mini-mental state examination; CSDD: Cornell Scale for Depression in Dementia; CMAI: Cohen–Mansfield Agitation Inventory; CRBRS: Care Recipient Behavior Rating Scale; TRR: test–retest reliability; SLUMS: Saint Louis University Mental Status Exam; OERS: Observed Emotion Rating Scale; EEG: electroencephalography; iEEG: intracranial electroencephalography; MRI: magnetic resonance imaging; fMRI: functional magnetic resonance imaging.
